# Preoperative albumin-to-fibrinogen ratio predicts severe postoperative complications in elderly gastric cancer subjects after radical laparoscopic gastrectomy

**DOI:** 10.1186/s12885-019-6143-x

**Published:** 2019-09-18

**Authors:** Xuexue You, Qun Zhou, Jie Song, Linguang Gan, Junping Chen, Huachun Shen

**Affiliations:** 1 Department of Anesthesiology, Jiangxi Maternal and Child Health Hospital, Nanchang, China; 2 Department of Anesthesiology, HwaMei Hospital, University of Chinese Academy of Sciences, No.41 Xibei Road, Haishu District, Ningbo, Zhejiang Province China

**Keywords:** Gastric cancer, Severe postoperative complications, Predictor, Albumin, Fibrinogen

## Abstract

**Background:**

A high prevalence of postoperative complications is closely associated with a worse short- and long-term outcome. This current study aimed to investigate potential risk factors including albumin-to-fibrinogen ratio (AFR) for severe postoperative complications (SPCs) in surgical gastric cancer (GC) patients.

**Methods:**

Elderly patients (≥65 years) with primary GC who underwent elective radical laparoscopic gastrectomy under general anesthesia were included. According to the Clavien–Dindo classification system, the severity of complications was assessed from Grade I to V and SPCs were defined as C-D Grade ≥ IIIa. The clinicopathological features, operative-associated characteristics, postoperative recovery and laboratory tests were compared between patients with or without SPCs. Receiver operating characteristic (ROC) curve analysis using Youden’s Index was established for determining the predictive value and cut-off threshold of AFR for SPCs. Binary univariate and multivariate logistic regression models were used to assess factors influencing SPCs.

**Results:**

A total of 365 elderly GC patients were finally included in the analysis, of which 52 (52/365, 14.2%) patients had developed SPCs within postoperative 30 days. Preoperative AFR level predicted SPCs in surgical GC patients with an AUC of 0.841, a sensitivity of 76.36% and a specificity of 80.77%, respectively (*P* < 0.001). The multivariate analysis revealed that a lower AFR level (OR: 1.94, 95% CI: 1.09–3.36, *P* = 0.017) and an older age (OR: 1.81, 95% CI: 1.06–3.04, *P* = 0.023) were two independent predictive factors for SPCs in surgical GC patients.

**Conclusions:**

Preoperative AFR level is a useful predictor for SPCs in elderly GC subjects after radical laparoscopic gastrectomy.

## Background

Gastric cancer (GC) is the fourth most common malignant neoplasm with an increasing incidence and it ranks second in cancer mortality worldwide [1]. Due to the high prevalence, recurrence rate and mortality rate, GC has become a significant global health problem [2]. As for resectable GC, surgical resection with systematic lymphadenectomy remains the standard treatment [3]. Advances in surgical techniques, instruments, and experiences have led to a corresponding decrease of postoperative complications, as well as improved outcomes [4]. However, a high prevalence of postoperative complications, with ranges from 14.3 to 34.0%, is closely associated with an increased economic burden, a prolonged hospital stay, a worse short- and long-term outcome [5]. Therefore, to improve the overall prognoses of GC patients, robust biomarkers for predicting severe postoperative complications (SPCs) after radical gastrectomy could help with the risk identification, follow-up facilitation and more aggressive postoperative care. Despite the significant improved perioperative managements, multidisciplinary therapeutic strategies and surgical techniques, a high prevalence of SPCs still remains to some extent [6].

Albumin (Alb), as a negative acute phase protein and a nutritional biomarker, usually decreases after surgery due to the surgery stress and increased capillary leakage [7]. Preoperative serum Alb is reported to be a predictive factor for postoperative recovery [8] and long-term survival [9] in GC patients. Another study has revealed that postoperative decrease of serum Alb expression can serve as a predictor for short-term complications in GC patients [10]. Preoperative low serum Alb expression was reported to be a potential risk factor for SPCs in elderly GC subjects [11]. Fibrinogen (Fib) is an essential protein for coagulation cascade as well as an acute-phase reaction protein in response to systemic inflammation [12]. Kanda et al. have indicated that Fib level is associated with tumor stage, metastasis, and outcomes in solid tumors, including GC [13]. Moreover, low preoperative Fib level is suggested as a potential risk factor for neurological complications after cardiac surgeries [14]. However, whether Alb or Fib can serve as a predictor for SPCs in GC patients still remains controversial. Alb-to-Fib ratio (AFR), a combination of Alb and Fib, has been reported to be a prognostic factor for non small-cell lung cancer patients [15, 16]. This study focused on the potential prognostic role of AFR for SPCs in GC patients.

## Methods

### Patients

This retrospective study was approved by the Medical Institutional Ethics Committee of Jiangxi province and our hospital. Elderly patients with primary GC who underwent elective radical laparoscopic gastrectomy under general anesthesia at the Department of anesthesiology, Jiangxi maternal and child health hospital from March 2014 to March 2018 were included. All the participants provided written informed consent. Inclusion criteria were described as follows: 1) elderly patients aged between 65 and 80 years; 2) diagnosed with primary GC which was supported by operative and pathological results; 3) undergoing systemic evaluation before the surgery including computed tomography (CT) image and 4) undergoing elective radical laparoscopic gastrectomy for the first time. The exclusion criteria were described as follows: 1) with tumor distant metastasis; 2) undergoing emergency operations due to complications (bowel obstruction, perforation, etc.) before the surgery; 3) with preoperative neoadjuvant treatment (radiotherapy or chemotherapy); 4) combined with other malignancies; 5) undergoing laparotomy or laparoscopic conversion to laparotomy; 6) with incomplete data. A total of 402 patients were initially enrolled and 37 patients were excluded according to the exclusion criteria (4 with tumor distant metastasis, 5 underwent emergency operations, 3 combined with other malignancies, 10 underwent laparotomy or laparoscopic conversion to laparotomy, 15 with data missed).

### Study design

The operative procedures for GC (the extent of gastrectomy and lymph node dissection) in this study was according to the Japanese Gastric Cancer Treatment Guidelines 2010 (ver. 3) [17]. All the enrolled patients were operated by the same experienced surgeons with the same perioperative managements. The pathological diagnosis was performed following the guidelines of the American Joint Committee on Cancer (AJCC) TNM Staging System for GC [18].

### Data collection

The following data were extracted and recorded from our database: 1) clinicopathological features, including age, gender, body mass index (BMI), American Society of Anesthesiologists (ASA) grade, comorbidities, smoking and drinking habits, abdominal surgery history, tumor location, histologic type, pathological type, tumor size and TNM stage; 2) operative-associated characteristics including extent of resection, operation time, estimated blood loss, intraoperative fluid utilization and perioperative blood transfusion; 3) postoperative recovery including time to first flatus, ambulation, first liquid intake and first soft intake; 4) laboratory tests.

### Assessment and definition of postoperative complications

The primary end point of this study was set as the occurrence of postoperative complications within postoperative 30 days [19]. According to the Clavien–Dindo classification system, the severity of complications was assessed from Grade I to V and SPCs were defined as C-D Grade ≥ IIIa [20]. If the patient had multiple complications, the grading was performed based on the most serious complication. Each patient was assessed for C-D grading by two independent experienced surgeons and divergences were solved by discussion. Enrolled patients were subdivided into SPCs group and non-SPCs group according to the presence of SPCs within postoperative 30 days.

### Laboratory tests

Fasting blood samples from each participant were obtained on 1 day before the operation. Blood cell analyses including hemoglobin (Hb), white blood cell (WBC), platelet (Plt) and hematocrit (Hct), biochemistry analyses including creatinine and urea were determined in the laboratory of our hospital. The serum expressions of inflammatory cytokines including tumor necrosis factor-α (TNF-α), C-reactive protein (CRP) and interleukin-6 (IL-6) were measured using the method of enzyme-linked immunesorbent assays (ELISA). The measurement procedures were performed according to the manufacturers’ instructions (R&D Systems, Minneapolis, MN, USA). AFR was calculated by Alb (g/L)/Fib (g/L) ratio.

### Statistical analysis

Statistical analysis was performed using SPSS 22.0 (SPSS Inc., IA, USA) and GraphPad Prism 5.0 (GraphPad Inc., CA, USA). All variables are expressed as means ± standard deviation (SD) or numbers with percentage (n, %). Continuous variables were compared using Mann–Whitney U test or Student t test, whereas categorical variables using Chi-square test or Fisher exact test as appropriate. Receiver operating characteristic (ROC) curve analysis using Youden’s Index was established for determining the predictive value and cut-off threshold of AFR for SPCs. Binary univariate and multivariate logistic regression models were used to assess factors influencing SPCs. Following univariate analysis, potential risk factors (*P* < 0.1) were selected for multivariate analysis using the multivariate logistic regression model with binary stepwise regression method. A two-sided *P* value of < 0.05 was considered statistically significant.

## Results

### Patient characteristics

According to the inclusion and exclusion criteria, 365 patients were enrolled in the analysis. The mean age of this study population was 73.1 years and the majority (275/365, 75.3%) were male patients. Among these 365 available participants, 52 were categorized into SPCs group with a prevalence of 14.2% (52/365) and the remaining 313 were categorized into non-SPCs group. The actual number and frequency of each complication in SPCs group are shown in Table 1. Of these postoperative events, pulmonary complications (*n* = 11, 3.0%), postoperative bleeding (*n* = 9, 2.5%), intra-abdominal infection (*n* = 8, 2.2%), bowel obstruction (*n* = 6, 1.6%), wound infection (*n* = 5, 1.4%) and anastomotic leakage (*n* = 4, 1.1%) are the most frequent. The demographic and clinical characteristics of enrolled GC patients associated with SPCs are shown in Table 2. As a result, those patients with an older age and a higher ASA grade were more likely to suffer SPCs (*P* < 0.05). The presence of preoperative comorbidities (hypertension and diabetes) was closely associated with an increased risk of SPCs (*P* = 0.013 and 0.019, respectively). The SPCs group experienced significantly longer operation time (*P* = 0.023), higher estimated blood loss (*P* = 0.012) and intraoperative fluid utilization (*P* = 0.007). No statistical differences were observed between SPCs and non-SPCs groups in gender, BMI, hyperlipidaemia, smoking and drinking habits, abdominal surgery history, tumor location, histologic type, pathological type, tumor size, TNM stage, extent of resection, perioperative blood transfusion, time to first flatus, ambulation, first liquid intake and first soft intake (*P* > 0.05).

**Table 1:** Number and frequency of SPCs

Complications	n (%)
Total	52
Pulmonary complications	11
Postoperative bleeding	9
Intra-abdominal infection	8
Bowel obstruction	6
Wound infection	5
Anastomotic leakage	4
Thrombosis	2
Heart failure	2
Others	5

*SPCs* Severe postoperative complications

**Table 2:** Demographic and clinical characteristics of GC patients with SPCs or not

Parameters	SPCs		*P*-value
	No (*n* = 313)	Yes (*n* = 52)	
Age (year)	72.3 ± 5.7	77.6 ± 6.3	< 0.001*
Gender, n (%)			0.449
Male	238	37	–
Female	75	15	–
BMI (kg/m^2^)	21.2 ± 2.1	20.9 ± 1.9	0.331
Comorbidities, n (%)			–
Hypertension	38	13	0.013*
Diabetes	27	10	0.019*
Hyperlipidaemia	22	7	0.122
Heavy drinkers, n (%)	26	7	0.230
Current smokers, n (%)	33	10	0.072
ASA grade, n (%)			0.015*
I/II	247	33	–
III/IV	66	19	–
Abdominal surgery history, n (%)	26	7	
Tumor location, n (%)			0.449
Cardia	32	6	–
Pylorus	201	28	–
Corpus	67	14	–
Total	13	4	–
Histologic type			0.177
Differentiated	221	30	–
Undifferentiated	33	8	–
Signet-ring cell carcinoma	59	14	–
Pathological type			0.210
Ulcerative	273	42	–
Non-ulcerative	40	10	–
Tumor size (cm)	4.3 ± 1.9	4.5 ± 2.1	0.489
Extent of resection			0.741
Distal gastrectomy	185	32	–
Total gastrectomy	128	20	–
T stage			0.876
Tis/T1/T2	130	21	–
T3/T4	183	31	–
N stage			0.952
N0	101	17	–
N1/N2/N3	212	35	–
TNM stage			0.654
I	77	11	–
II	89	13	–
III	147	28	–
Operation time (min)	234.6 ± 31.3	245.7 ± 38.2	0.023*
Estimated blood loss (mL)	187.9 ± 78.8	217.2 ± 70.6	0.012*
Intraoperative fluid utilization (mL)	1910.3 ± 245.3	2014.3 ± 306.4	0.007*
Perioperative blood transfusion, n (%)	105	16	0.694
Time to first flatus (d)	2.9 ± 0.7	3.0 ± 0.9	0.362
Time to ambulation (d)	2.1 ± 0.7	2.2 ± 0.6	0.332
Time to first liquid intake (d)	3.9 ± 1.1	4.0 ± 1.3	0.555
Time to first soft intake (d)	5.4 ± 1.3	5.3 ± 1.5	0.616

*P*-values were calculated by Chi-square test, Fisher exact test, Mann-Whitney U or t test

*GC* Gastric cancer, *SPCs* Severe postoperative complications, *BMI* Body mass index, *ASA* American Society of Anesthesiologists

*P value< 0.05

### Laboratory tests and SPCs

Table 3 shows the results of preoperative laboratory tests in surgical patients with or without SPCs. Patients who suffered SPCs had higher preoperative expressions of CRP (*P* = 0.012) and TNF-α (*P* = 0.016) than those who did not. Moreover, those patients with a lower preoperative AFR level were more likely to develop SPCs (*P* < 0.001). There were no significant differences in blood cell analyses, IL-6, Alb, Fib, creatinine and urea between the two groups (*P* > 0.05).

**Table 3:** Laboratory tests and SPCs in GC patients

Preoperative laboratory tests	SPCs		*P*-value
	No (*n* = 313)	Yes (*n* = 52)	
Hb (g/L)	117.5 ± 7.5	116.4 ± 8.4	0.337
Plt (×10^9^/L)	207.6 ± 41.2	214.3 ± 49.7	0.293
WBC (×10^9^/L)	7.1 ± 2.2	6.9 ± 1.9	0.537
Hct	0.43 ± 0.07	0.42 ± 0.06	0.332
CRP (mg/L)	6.3 ± 3.1	7.5 ± 3.5	0.012*
IL-6 (pg/mL)	15.7 ± 7.1	16.3 ± 6.6	0.569
TNF-α (nmol/L)	7.6 ± 1.9	8.3 ± 2.1	0.016*
Creatinine (mmol/L)	83.1 ± 17.3	82.6 ± 18.4	0.848
Urea (mmol/L)	6.4 ± 1.8	6.2 ± 1.7	0.455
Albumin (g/L)	39.2 ± 5.5	37.9 ± 6.2	0.122
Fibrinogen (mg/dL)	3.6 ± 1.3	3.9 ± 1.5	0.134
AFR	9.9 ± 2.2	7.4 ± 2.1	< 0.001*

*P*-values were calculated by Mann-Whitney U or t test

*GC* Gastric cancer, *SPCs* Severe postoperative complications, *Hb* Hemoglobin, *Plt* Platelet, *WBC* White blood cell, *Hct* Hematocrit, *CRP* C-reactive protein, *IL-6* Interleukin-6, *TNF-α* Tumor necrosis factor-α, *AFR* Albumin-to-fibrinogen ratio

*P value< 0.05

### AFR and SPCs

**Fig. 1:**
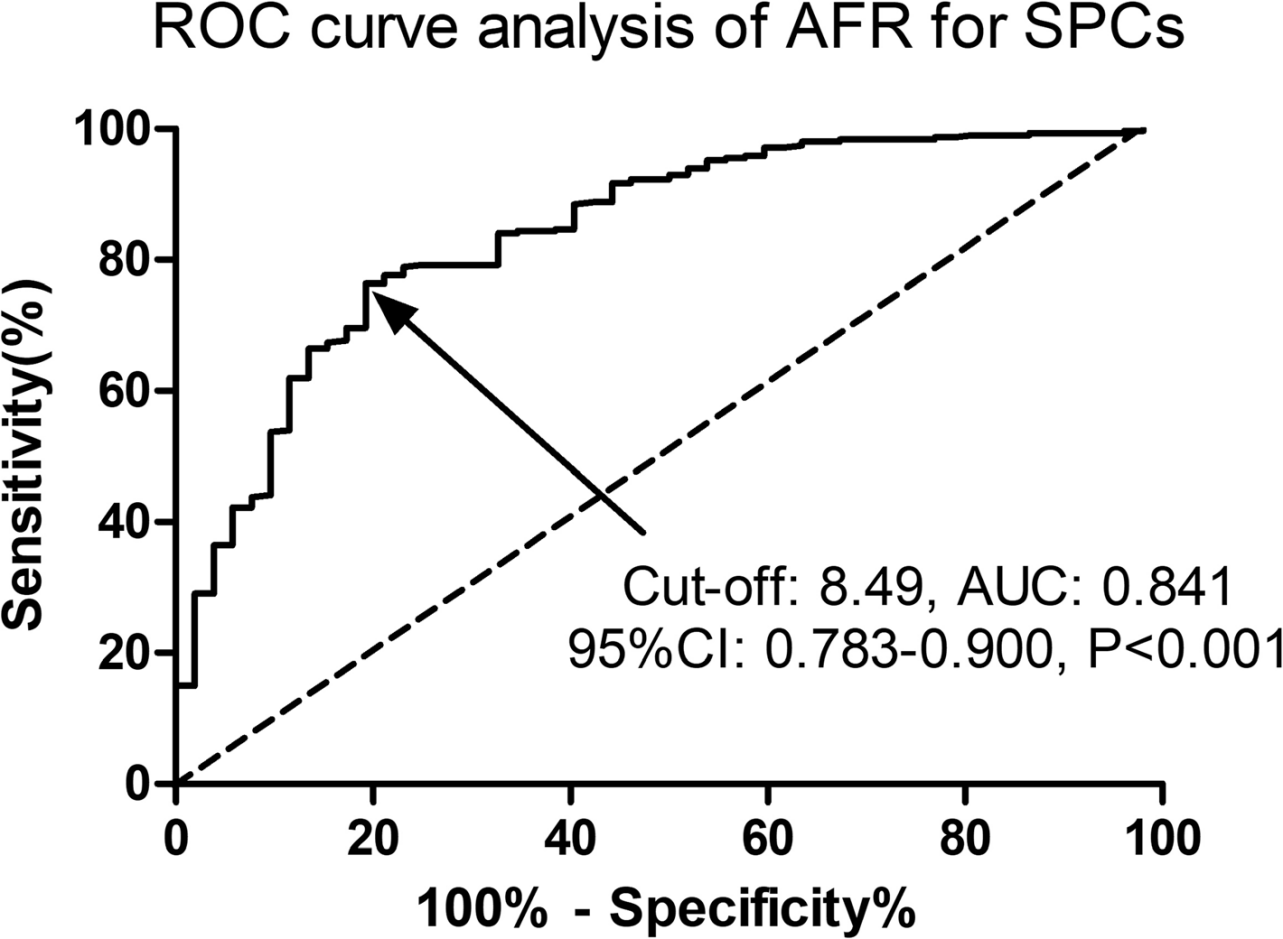
Predictive value of AFR for SPCs in GC patients by ROC curve analysis. The results indicated preoperative AFR as a potential predictive factor for SPCs in GC patients with an AUC of 0.841, 95%CI of 0.783–0.900, a cut-off value of 8.49, a sensitivity of 76.36% and a specificity of 80.77%, respectively (*P* < 0.001). AFR, albumin-to-fibrinogen ratio; SPCs, severe postoperative complications; GC, gastric cancer; ROC, receiver operating characteristic; AUC, the area under the curve; CI, confidence interval

ROC curve analysis was performed to evaluate the predictive value of AFR for SPCs in GC patients. As illustrated in Fig. 1, preoperative AFR level predicted SPCs in surgical GC patients with an AUC of 0.841, a sensitivity of 76.36% and a specificity of 80.77%, respectively (*P* < 0.001). Furthermore, an AFR value of 8.49 was set as the optimal cut-off threshold for SPCs based on the Youden’s Index. Enrolled patients were then subdivided by AFR based on the cut-off value, high-AFR group (AFR>8.49) and low-AFR group (AFR ≤ 8.49).

### Risk factors for SPCs

All potential risk factors (*P* < 0.05 in Tables 2 and 3, *n* = 10 in this study, see Table 4) were enrolled in the univariate and multivariate logistic regression analyses. In the univariate analysis, five risk factors with *P* values < 0.1 (age, diabetes, operation time, CRP and AFR) were selected for multivariate analysis. The multivariate analysis revealed that a lower AFR level (OR: 1.94, 95% CI: 1.09–3.36, *P* = 0.017) and an older age (OR: 1.81, 95% CI: 1.06–3.04, *P* = 0.023) were two independent predictive factors for SPCs in surgical GC patients.

**Table 4:** Risk factors for SPCs by univariate and multivariate logistic regression analyses

Variables	Univariate		Multivariate	
	OR (95%CI)	*P* value	OR (95%CI)	*P* value
Age (≥74 vs < 74)	2.22 (1.37–3.58)	0.005*	1.81 (1.06–3.04)	0.023*
Hypertension (yes vs no)	1.41 (0.83–2.39)	0.21		
Diabetes (yes vs no)	1.72 (1.04–2.88)	0.031*	1.29 (0.78–2.19)	0.41
ASA grade (I/II vs III/IV)	0.96 (0.59–1.51)	0.81		
Operation time (high vs low)	1.71 (1.03–2.85)	0.029*	1.54 (0.85–2.63)	0.14
Estimated blood loss (high vs low)	0.94 (0.51–1.79)	0.85		
Intraoperative fluid utilization (high vs low)	0.92 (0.53–1.58)	0.79		
Preoperative CRP (high vs low)	1.83 (1.11–3.08)	0.024*	1.37 (0.79–2.33)	0.25
Preoperative TNF-α (high vs low)	1.15 (0.73–1.77)	0.42		
AFR (≤8.49 vs >8.49)	2.54 (1.52–4.22)	0.001*	1.94 (1.09–3.36)	0.017*

The high vs low levels were categorized using the median value as the cut-off value

*SPCs* Severe postoperative complications, *ASA* American Society of Anesthesiologists, *CRP* C-reactive protein, *TNF-α* Tumor necrosis factor-α, *AFR* Albumin-to-fibrinogen ratio, *OR* Odds ratio, *CI* Confidence interval

*P value< 0.05

## Discussion

In this present study, we observed that preoperative AFR level and age were two independent predictive factors for SPCs in GC patients undergoing elective radical laparoscopic gastrectomy. Our study reported a prevalence of SPCs of 14.2% in surgical GC patients, which was a little higher than 10.2% by Zhang et al. [21] and 8.1% by Kang et al. [11]. Another study by Fukuda et al. has reported a prevalence of 13.2% [22], which is quite in accordance with our results. In our consideration, the different sample sizes, age ranges, inclusion and exclusion criteria, SPCs evaluation deviations and some missed data might be possible explanations for the different results.

It is well known that diabetes closely correlates with postoperative complications. Patients who had comorbidities of diabetes before surgery were associated with a high risk of major postoperative complications after reconstructive microsurgery for head and neck cancer [23]. Furthermore, Saji et al. indicated a comprehensive risk scoring system, which included diabetes mellitus as a component, capable of predicting SPCs in patients with medically operable lung cancer [24]. A previous study by Sung et al. reported that a long operation time (> 3 h) was an independent risk factor for severe and overall postoperative complications, as well as poor surgical outcomes [11]. As reported by recent studies, CRP is suggested to be a valid predictor of postoperative complications after various operations, such as minimally invasive colorectal resection [25], minimally invasive esophagectomy [26] and major noncardiac surgery [27]. A randomized controlled trial has also proved the significantly predictive value of CRP for surgical site infection [28]. Our univariate analyses showed that five variables (age, diabetes, operation time, CRP and AFR) might be potential risk factors for SPCs. However, the results from our multivariate analyses only supported age and AFR as two independent risk factors for SPCs. The different sample sizes, operation types, perioperative managements may explain the different conclusions.

Accumulating evidence has demonstrated that aging is an independent risk factor for postoperative complications following various operation types, including pancreatic resection [29], laparoscopic gastrectomy [30], and robotic-assisted pulmonary lobectomies [31]. As expected, our results also supported an older age as an independent risk factor for SPCs. Several studies have indicated that older age is associated with high postoperative morbidity and mortality rates due to increased preoperative comorbidities [32]. We consider that the age-associated gradual loss of reserve capacity (e.g. circulatory, immune system changes) [33] and more preexisting diseases [34] may be possible mechanisms for its predictive value for SPCs in this study. However, there is still no consensus with respect to the cut-offs of ages among the published studies [29].

Alb is a sensitive biomarker for nutritional status evaluation and an acute-phase protein in response to systemic inflammation [35]. Alb expressions are recommended to be a reliable prognostic factor in patients with cancers [36]. Fib, which is synthesized by liver, is a crucial component of blood coagulation system via promoting platelet aggregation. Moreover, Fib is reported to be an important biomarker reflecting systemic inflammation [37] and it serves as a candidate prognostic biomarker in patients with non-small cell lung cancer (NSCLC) [38]. AFR, a ratio of Alb-to-Fib, combines these two biomarkers and amplifies the sensitivity for evaluating inflammation and nutrition status. The combination of Alb and Fib is superior to the single Alb and Fib and it has been widely recommended as a prognostic factor in various models, e.g. acute ST-segment elevation myocardial infarction (STEMI) [39], operable NSCLC [15], and operable soft tissue sarcoma [40]. Preoperative low serum Alb is reported to be an indicator for postoperative complications and mortality in patients undergoing transcatheter aortic valve replacement [41]. Furthermore, early decrease in Alb is a significant predictor for SPCs in colorectal cancer patients undergoing curative laparoscopic surgery [42]. Previous studies have also indicated Fib as an early marker of postoperative complications after laparoscopic sleeve gastrectomy in morbidly obese patients [43] or total joint arthroplasty [44]. This present study was the first to indicate preoperative AFR as an independent risk factor for SPCs in GC patients after radical laparoscopic gastrectomy. The close association between inflammation and SPCs might be a possible mechanism.

This study had some certain limitations. First, this is a single-center study with the retrospective nature, so selection bias is inevitable. An independent prospective cohort is required to validate a definitive conclusion regarding clinical application of AFR and its optimal cutoff for SPCs prediction in surgical GC patients. Second, this study only takes preoperative Alb and Fib into consideration, whether postoperative levels have the predictive value remains unclear. Furthermore, the involved mechanisms for this study remain uncertain. A multi-center study with larger sample size was required to validate the prognostic role of AFR in GC patients. Furthermore, whether the interventions of AFR (e.g. improve the nutritional status, hypoproteinemia, coagulation function) could improve the outcomes in GC patients and decrease SPCs remains unclear.

## Conclusions

To the best of our knowledge, this study firstly highlighted that preoperative AFR and age were two independent risk factors for SPCs in elderly surgical GC patients. Of course, our results do not support the delaying of elective surgery according to the preoperative AFR values. Instead, the situations with potential development of SPCs should be considered and intensively cared.

## Data Availability

Please contact the author Huachun Shen (shenhuachun_nb@sina.com) upon reasonable requests.

## Abbreviations

AFR: Albumin-to-fibrinogen ratio
ASA: American Society of Anesthesiologists
BMI: Body mass index
CI: Confidence interval
CRP: C-reactive protein
GC: Gastric cancer
Hb: Hemoglobin
Hct: Hematocrit
IL-6: Interleukin-6
NSCLC: Non-small cell lung cancer
OR: Odds ratio
Plt: Platelet
SPCs: Severe postoperative complications;
STEMI: ST-segment elevation myocardial infarction
TNF-α: Tumor necrosis factor-α
WBC: White blood cell

## References

[1] Torre LA, Bray F, Siegel RL, Ferlay J, Lortet-Tieulent J, Jemal A (2015). Global cancer statistics, 2012. CA Cancer J Clin.

[2] Van Cutsem E, Sagaert X, Topal B, Haustermans K, Prenen H (2016). Gastric cancer. Lancet.

[3] Sasahara M, Kanda M, Ito S, Mochizuki Y, Teramoto H, Ishigure K, et al. The preoperative prognostic nutritional index predicts short-term and long-term outcomes of patients with stage II/III gastric cancer: analysis of a multi-institution dataset. Dig Surg. 2019:1–10. 10.1159/000497454. 10.1159/000497454 30840952

[4] Yau GL, Silva PS, Arrigg PG, Sun JK (2018). Postoperative complications of pars Plana vitrectomy for diabetic retinal disease. Semin Ophthalmol.

[5] Imamura H, Kurokawa Y, Kawada J, Tsujinaka T, Takiguchi S, Fujiwara Y (2011). Influence of bursectomy on operative morbidity and mortality after radical gastrectomy for gastric cancer: results of a randomized controlled trial. World J Surg.

[6] Papamichael D, Audisio RA, Glimelius B, de Gramont A, Glynne-Jones R, Haller D (2015). Treatment of colorectal cancer in older patients: international society of geriatric oncology (SIOG) consensus recommendations 2013. Ann Oncol.

[7] Fleck A, Raines G, Hawker F, Trotter J, Wallace PI, Ledingham IM (1985). Increased vascular permeability: a major cause of hypoalbuminaemia in disease and injury. Lancet.

[8] Toiyama Y, Yasuda H, Ohi M, Yoshiyama S, Araki T, Tanaka K (2017). Clinical impact of preoperative albumin to globulin ratio in gastric cancer patients with curative intent. Am J Surg.

[9] Yamashita K, Ushiku H, Katada N, Hosoda K, Moriya H, Mieno H (2015). Reduced preoperative serum albumin and absence of peritoneal dissemination may be predictive factors for long-term survival with advanced gastric cancer with positive cytology test. Eur J Surg Oncol.

[10] Liu ZJ, Ge XL, Ai SC, Wang HK, Sun F, Chen L (2017). Postoperative decrease of serum albumin predicts short-term complications in patients undergoing gastric cancer resection. World J Gastroenterol.

[11] Kang SC, Kim HI, Kim MG (2016). Low serum albumin level, male sex, and total gastrectomy are risk factors of severe postoperative complications in elderly gastric cancer patients. J Gastric Cancer.

[12] Kijima T, Arigami T, Uchikado Y, Uenosono Y, Kita Y, Owaki T (2017). Combined fibrinogen and neutrophil-lymphocyte ratio as a prognostic marker of advanced esophageal squamous cell carcinoma. Cancer Sci.

[13] Kanda M, Tanaka C, Kobayashi D, Mizuno A, Tanaka Y, Takami H (2017). Proposal of the coagulation score as a predictor for short-term and long-term outcomes of patients with resectable gastric cancer. Ann Surg Oncol.

[14] Guan X, Gong M, Wang X, Zhu J, Liu Y, Sun L (2018). Low preoperative fibrinogen level is risk factor for neurological complications in acute aortic dissection. Medicine (Baltimore).

[15] Ying J, Zhou D, Gu T, Huang J, Liu H (2019). Pretreatment albumin/fibrinogen ratio as a promising predictor for the survival of advanced non small-cell lung cancer patients undergoing first-line platinum-based chemotherapy. BMC Cancer.

[16] Li SQ, Jiang YH, Lin J, Zhang J, Sun F, Gao QF (2018). Albumin-to-fibrinogen ratio as a promising biomarker to predict clinical outcome of non-small cell lung cancer individuals. Cancer Med.

[17] Japanese Gastric Cancer A (2011). Japanese gastric cancer treatment guidelines 2010 (ver. 3). Gastric Cancer.

[18] In H, Solsky I, Palis B, Langdon-Embry M, Ajani J, Sano T (2017). Validation of the 8th edition of the AJCC TNM staging system for gastric cancer using the national cancer database. Ann Surg Oncol.

[19] Cao X, Zhao G, Yu T, An Q, Yang H, Xiao G (2017). Preoperative prognostic nutritional index correlates with severe complications and poor survival in patients with colorectal cancer undergoing curative laparoscopic surgery: a retrospective study in a single Chinese institution. Nutr Cancer.

[20] Clavien PA, Barkun J, de Oliveira ML, Vauthey JN, Dindo D, Schulick RD (2009). The Clavien-Dindo classification of surgical complications: five-year experience. Ann Surg.

[21] Zhang WT, Lin J, Chen WS, Huang YS, Wu RS, Chen XD (2018). Sarcopenic obesity is associated with severe postoperative complications in gastric cancer patients undergoing gastrectomy: a prospective study. J Gastrointest Surg.

[22] Fukuda Y, Yamamoto K, Hirao M, Nishikawa K, Nagatsuma Y, Nakayama T (2016). Sarcopenia is associated with severe postoperative complications in elderly gastric cancer patients undergoing gastrectomy. Gastric Cancer.

[23] Lo SL, Yen YH, Lee PJ, Liu CC, Pu CM (2017). Factors influencing postoperative complications in reconstructive microsurgery for head and neck cancer. J Oral Maxillofac Surg.

[24] Saji H, Ueno T, Nakamura H, Okumura N, Tsuchida M, Sonobe M (2018). A proposal for a comprehensive risk scoring system for predicting postoperative complications in octogenarian patients with medically operable lung cancer: JACS1303. Eur J Cardiothorac Surg.

[25] Pedrazzani C, Moro M, Mantovani G, Lazzarini E, Conci S, Ruzzenente A (2017). C-reactive protein as early predictor of complications after minimally invasive colorectal resection. J Surg Res.

[26] Miki Y, Toyokawa T, Kubo N, Tamura T, Sakurai K, Tanaka H (2017). C-reactive protein indicates early stage of postoperative infectious complications in patients following minimally invasive esophagectomy. World J Surg.

[27] Vasunilashorn SM, Dillon ST, Inouye SK, Ngo LH, Fong TG, Jones RN (2017). High C-reactive protein predicts delirium incidence, duration, and feature severity after major noncardiac surgery. J Am Geriatr Soc.

[28] Mujagic E, Marti WR, Coslovsky M, Zeindler J, Staubli S, Marti R (2018). The role of preoperative blood parameters to predict the risk of surgical site infection. Am J Surg.

[29] Chen YT, Ma FH, Wang CF, Zhao DB, Zhang YW, Tian YT (2018). Elderly patients had more severe postoperative complications after pancreatic resection: a retrospective analysis of 727 patients. World J Gastroenterol.

[30] Yu J, Hu J, Huang C, Ying M, Peng X, Wei H (2013). The impact of age and comorbidity on postoperative complications in patients with advanced gastric cancer after laparoscopic D2 gastrectomy: results from the Chinese laparoscropic gastrointestinal surgery study (CLASS) group. Eur J Surg Oncol.

[31] Kass KS, Velez-Cubian FO, Zhang WW, Toosi K, Tanvetyanon T, Rodriguez KL (2017). Effect of advanced age on peri-operative outcomes after robotic-assisted pulmonary lobectomy: retrospective analysis of 287 consecutive cases. J Geriatr Oncol.

[32] Gretschel S, Estevez-Schwarz L, Hunerbein M, Schneider U, Schlag PM (2006). Gastric cancer surgery in elderly patients. World J Surg.

[33] Ritz P (2000). Physiology of aging with respect to gastrointestinal, circulatory and immune system changes and their significance for energy and protein metabolism. Eur J Clin Nutr.

[34] Polanczyk CA, Marcantonio E, Goldman L, Rohde LE, Orav J, Mangione CM (2001). Impact of age on perioperative complications and length of stay in patients undergoing noncardiac surgery. Ann Intern Med.

[35] Artigas A, Wernerman J, Arroyo V, Vincent JL, Levy M (2016). Role of albumin in diseases associated with severe systemic inflammation: pathophysiologic and clinical evidence in sepsis and in decompensated cirrhosis. J Crit Care.

[36] Gupta D, Lis CG (2010). Pretreatment serum albumin as a predictor of cancer survival: a systematic review of the epidemiological literature. Nutr J.

[37] Jensen T, Kierulf P, Sandset PM, Klingenberg O, Joo GB, Godal HC (2007). Fibrinogen and fibrin induce synthesis of proinflammatory cytokines from isolated peripheral blood mononuclear cells. Thromb Haemost.

[38] Sheng L, Luo M, Sun X, Lin N, Mao W, Su D (2013). Serum fibrinogen is an independent prognostic factor in operable nonsmall cell lung cancer. Int J Cancer.

[39] Zhao Y, Yang J, Ji Y, Wang S, Wang T, Wang F (2019). Usefulness of fibrinogen-to-albumin ratio to predict no-reflow and short-term prognosis in patients with ST-segment elevation myocardial infarction undergoing primary percutaneous coronary intervention. Heart Vessels.

[40] Liang Y, Wang W, Que Y, Guan Y, Xiao W, Fang C (2018). Prognostic value of the fibrinogen/albumin ratio (FAR) in patients with operable soft tissue sarcoma. BMC Cancer.

[41] Gassa A, Borghardt JH, Maier J, Kuhr K, Michel M, Ney S (2018). Effect of preoperative low serum albumin on postoperative complications and early mortality in patients undergoing transcatheter aortic valve replacement. J Thorac Dis.

[42] Wang Y, Wang H, Jiang J, Cao X, Liu Q (2018). Early decrease in postoperative serum albumin predicts severe complications in patients with colorectal cancer after curative laparoscopic surgery. World J Surg Oncol.

[43] Ruiz-Tovar J, Munoz JL, Gonzalez J, Garcia A, Ferrigni C, Jimenez M (2017). C-reactive protein, fibrinogen, and procalcitonin levels as early markers of staple line leak after laparoscopic sleeve gastrectomy in morbidly obese patients within an enhanced recovery after surgery (ERAS) program. Surg Endosc.

[44] Oelsner WK, Engstrom SM, Benvenuti MA, An TJ, Jacobson RA, Polkowski GG (2017). Characterizing the acute phase response in healthy patients following total joint arthroplasty: predictable and consistent. J Arthroplast.

